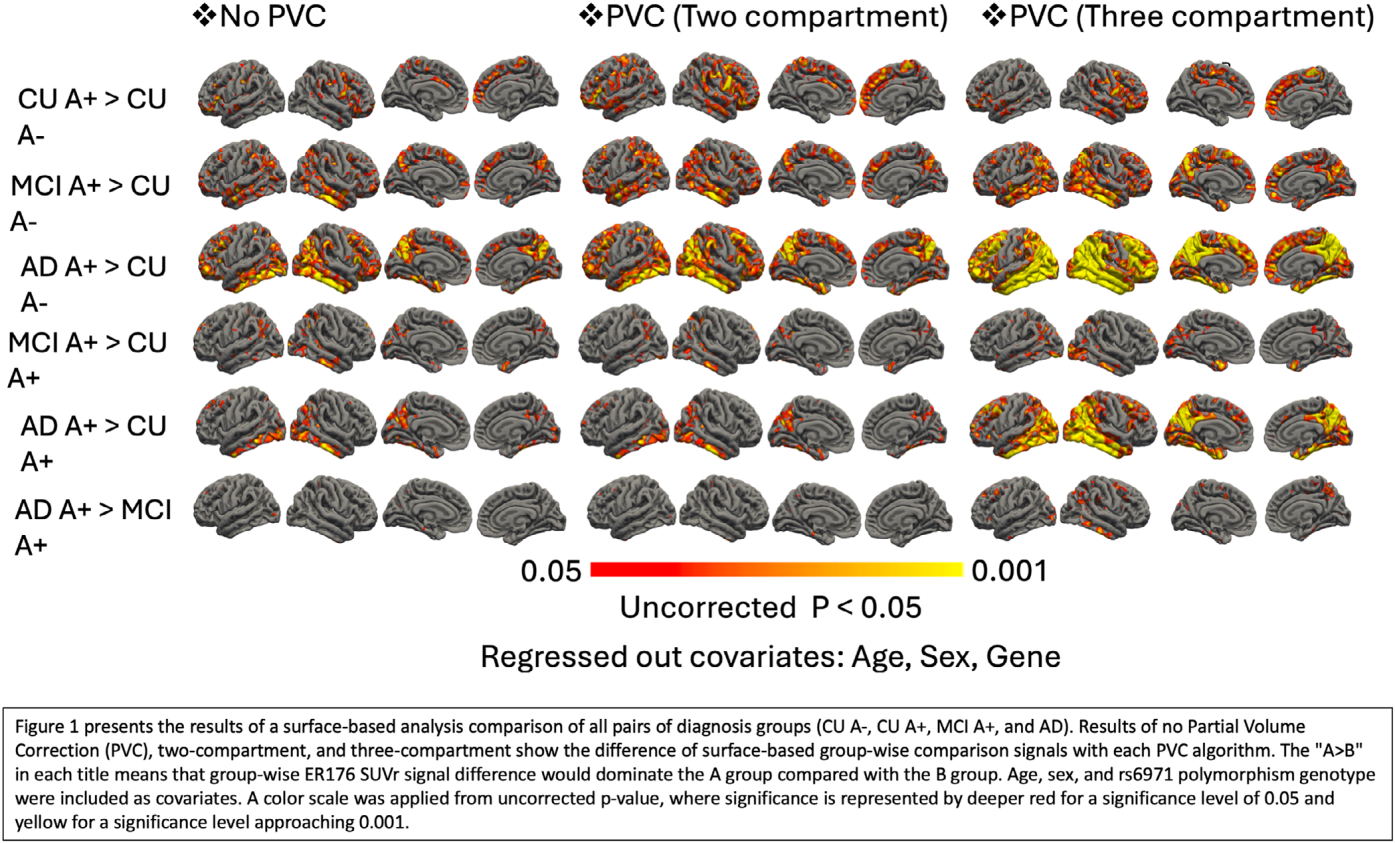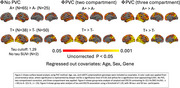# Assessing neuroinflammation in Alzheimer's disease: A TSPO‐PET study using ER176 and its correlation with amyloid and tau pathology

**DOI:** 10.1002/alz70856_106023

**Published:** 2026-01-09

**Authors:** Mahathi Kandimalla, David N Jacobson, Seokbeen Lim, Jeyeon Lee, Hoon‐Ki Min, Marin E Nycklemoe, Hugo Botha, Jonathan Graff‐Radford, David T. Jones, Prashanthi Vemuri, Kejal Kantarci, David S. Knopman, Clifford R. Jack, Ronald Petersen, Val J Lowe

**Affiliations:** ^1^ Mayo Clinic, Rochester, MN, USA; ^2^ Hanyang University, Seoul, Korea, Republic of (South); ^3^ Department of Radiology, Mayo Clinic, Rochester, MN, USA; ^4^ Department of Neurology, Mayo Clinic, Rochester, MN, USA

## Abstract

**Background:**

Neuroinflammation plays a crucial role in neuronal damage, synaptic dysfunction, and impaired neurogenesis, significantly contributing to Alzheimer's Disease (AD) progression. To address this, we assessed ER176's effectiveness in detecting neuroinflammation in cognitively unimpaired (CU) A–, CU A+, mild cognitive impairment (MCI), and AD participants, with a particular focus on their amyloid and tau status. This research aims to help identify suitable candidates for specialized treatment when adversely impacted by neuroinflammation.

**Method:**

Participants from the Mayo Clinic Study of Aging were recruited for this study. The study included four participant groups: CU A– (*n* = 25), CU A+ (*n* = 25), MCI A+ (*n* = 25), and AD (*n* = 15). We explored the variations in neuroinflammation PET results among different groups.

**Result:**

When compared to CU A–, both CU A+ and MCI A+ groups (Figure 1) showed greater regional neuroinflammation. AD exhibited more widely affected regions with neuroinflammation (temporal, occipital, and parietal lobes) than CU A‐ over the PVC spectrum (Figure 1). Minimally more neuroinflammation was seen (temporal lobes) in MCI A+ than CU A+ (Figure 1). AD had more widespread regional neuroinflammation vs CU A+ (Figure 1) than in MCI A+ vs CU+. The least difference in neuroinflammation is seen in MCI A+ vs AD (Figure 1). For A+ vs A‐, A+ showed more widespread neuroinflammation (Figure 2). T+ (tau>=1.29) vs T‐ (tau<1.29) showed more neuro inflammation with T+ (Figure 2). Comparisons varied modestly in significance level with PVC method (Figures 1 and 2).

**Conclusion:**

The findings demonstrate that neuroinflammatory changes are associated with AD pathology. Even at early stages, neuroinflammation is associated with CU+. As the condition progresses, neuroinflammation extends to more brain regions and is associated with increased A+ and T+ pathology in MCI A+ and AD. These results suggest an early and persistent role of neuroinflammation in the progression AD pathology, indicating that therapy options could include early anti‐inflammatory intervention, even prior to clinical symptoms, along with treatment during clinical manifestations. PVC methods used may provide somewhat variable results.